# Gentiopicroside—An Insight into Its Pharmacological Significance and Future Perspectives

**DOI:** 10.3390/cells13010070

**Published:** 2023-12-29

**Authors:** Lemonia Antoniadi, Magdalena Bartnik, Apostolis Angelis, Anna Wawruszak, Maria Halabalaki, Wirginia Kukula-Koch, Leandros A. Skaltsounis

**Affiliations:** 1 Division of Pharmacognosy and Natural Products Chemistry, Department of Pharmacy, National and Kapodistrian University of Athens, Panepistimioupoli Zografou, 15771 Athens, Greece; monikaant@pharm.uoa.gr (L.A.); aangjel@pharm.uoa.gr (A.A.); mariahal@pharm.uoa.gr (M.H.); skaltsounis@pharm.uoa.gr (L.A.S.); 2 Department of Pharmacognosy with Medicinal Plants Garden, Medical University of Lublin, 1 Chodźki Str., 20-093 Lublin, Poland; mbartnik65@gmail.com; 3 Department of Biochemistry and Molecular Biology, Medical University of Lublin, 1 Chodźki Str., 20-093 Lublin, Poland

**Keywords:** gentiopicroside, *Gentiana*, Gentianaceae, pharmacological activity, *in vitro*, *in vivo*, molecular mechanism of bioactivity, health benefits, semisynthetic derivatives of gentiopicroside

## Abstract

Gentiopicroside (GPS) is a leading component of several plant species from the Gentianaceae botanical family. As a compound with plenty of biological activities and a component of herbal drugs, GPS has an important role in the regulation of physiological processes in humans. The results of recently published scientific studies underline a meaningful role of this molecule as an active factor in metabolic pathways and mechanisms, which may have an influence in the treatment of different diseases, including digestive tract disorders, malignant changes, neurological disorders, microbial infections, bone formation disorders, inflammatory conditions, and others. This review aims to collect previously published reports on the biological properties of GPS as a single compound that were confirmed by *in vitro* and *in vivo* studies, and to draw attention to the newly discovered role of this bitter-tasting secoiridoid. Thanks to these properties, the research on this substance could be revisited.

## 1. Introduction

**Figure 1:**
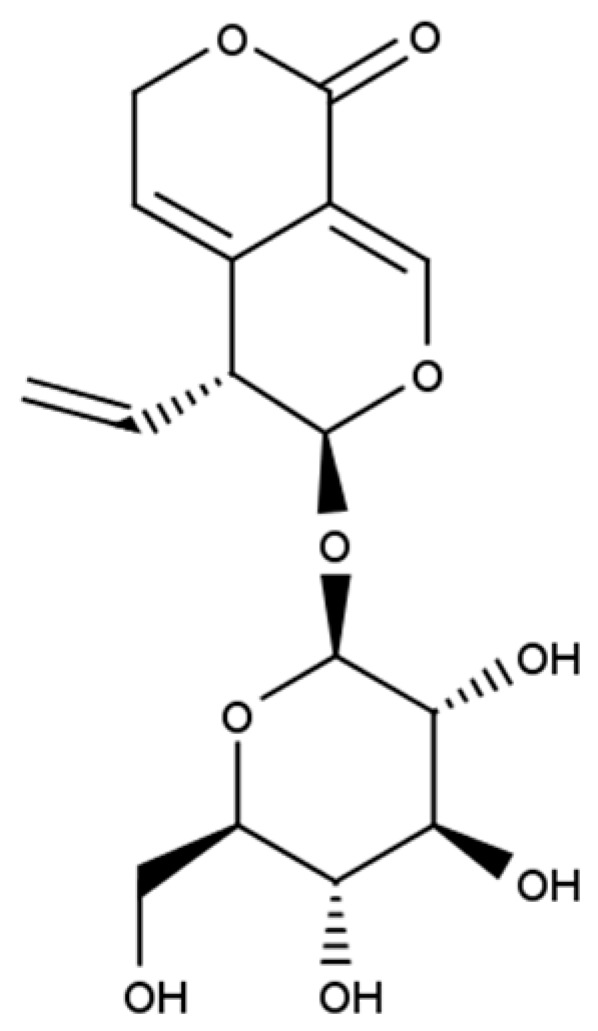
Chemical structure of gentiopicroside.

It is generally believed that medicines (including natural ones) do not have to taste good, but they must be good for our health. The least pleasant taste for many people is the bitter taste of both food and medicines. Bitter-tasting plant compounds represent many different chemical structures, including alkaloids, polyphenolic compounds (coumarins, flavonoids), glucosinolates, and terpenoids. Belonging to the last group, secoiridoid glucoside gentiopicroside (gentiopicrin, GPS, CAS No 20831-76-9; Figure 1) occurs mainly in the Gentianales order, in the botanical family Gentianaceae [1]. Interestingly, GPS has also been found in plant species from the other botanical families, including *Aster auriculatus* Franch (Asteraceae) [2], *Artocarpus heterophyllus* Lam. (Moraceae) [3]), and *Cephalaria kotschyi* (Dipsaceae) [4]. In the Gentianaceae family, which contains about 400 species that are native to Europe, Asia, and North America, GPS is present in the genera *Swertia*, *Centaurium*, and *Gentiana* [5].

Gentian has been used in natural medicine for centuries, including traditional Chinese medicine (TCM) and Ayurvedic medicine. Plants of the *Gentiana* genus are also listed in many modern Pharmacopoeias, including the 10th edition of the European Pharmacopoeia, where a bitterness index of not less than 10,000 is required for the root of *Gentiana lutea* L. (compared to the bitterness index of quinine hydrochloride of 200,000) [6]. In this valuable medicinal plant, gentiopicroside is one of the best-known active substances and was first isolated from this plant source in 1862 [1]. Since then, the secoiridoid has been extensively studied for its biological activity in various *in vitro* and *in vivo* models, as well as in a few clinical trials. GPS was found to be non-toxic at the doses used and relatively stable, although it is not readily bioavailable.

In this review, we intend to analyze and highlight the pharmacological potential of GPS as a biologically active compound of natural origin. Based on the available literature data, this article will discuss *in vitro* and *in vivo* experiments with GPS as an active metabolite isolated from natural sources and tested as a single compound (this review will not consider the effects of whole plant extracts containing GPS).

## 2. Materials and Methods

The articles and book chapters used to prepare this review manuscript were collected from the scientific databases like Scopus, PubMed, the Web of Science, Science Direct, and SciFinder CAS. To collect the data, the following keywords and their combinations were entered into the search engines: “gentiopicroside” or “gentiopicrin” and “*in vitro* activity”, “*in vivo* activity”, “clinical studies”, “animal studies”, “T2R receptors”, “toxicity”, and “occurrence”. The authors organized the literature search results into specific sections, taking into account the results related to our main goal, as previously declared.

## 3. Bioavailability, Biotransformation, and Stability of GPS 

GPS is an ingredient of plant drugs that are prepared from different gentian species and used as natural medicines (in Chinese medicine for more than 200 years) to improve digestion, for pain relief, and as a treatment of rheumatism because of its anti-inflammatory, antioxidant, or analgesic effects [5]. Bearing in mind GPS-containing preparations, it can be concluded that GPS is most often administered orally, and because of this fact, it undergoes the typical ADME processes, characterizing the transport of a substance in a living organism, namely the liberation, absorption, distribution, metabolism, and excretion. 

Some GPS metabolites were determined by Zeng et al. in their UPLC/Q-TOF MS-based study on gentiopicroside isolated from *Gentiana scabra* [7]. The authors proposed a possible metabolic pathway of GPS that was induced by ß-glucosidase in their *in vitro* assay. According to the authors, hydrolysis was an initial step to trigger biotransformation and produce an intermediate aglycone, which was next converted to four metabolites (M1–M4) via isomerization, reduction, and oxidation reactions. M1 (identified as gentiopicral) and M2 (identified as erythrocentaurin) were the main metabolic products formed by means of different isomerization paths at the hemiacetal part of aglycone. Metabolite M3 was proposed to be the lactone ring open metabolite of M, and M4 was identified as 5-(hydroxymethyl)-1H-isochromen-1-one. 

**Figure 2:**
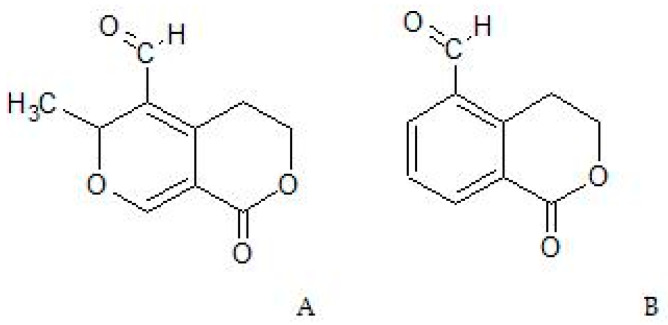
Structures of two main metabolites of GPS: gentiopicral (**A**) and erythrocentaurin (**B**).

The formation of GPS metabolites was attributed to the intestinal flora. The anaerobic incubation of the compound with each one of 24 strains of bacteria isolated from human feces resulted in the transformation of GPS to at least five metabolites in different ratios. The products of bacterial metabolism were extracted with ethyl acetate and analyzed via TLC–densitometry by El-Sedawy et al. [8]. In their study, GPS was hydrolyzed to deliver an unstable aglycone in a hemiacetal form that was converted to two types of compounds: isochroman and pyrano[3,4-*c*]pyran derivatives. The aldehydes of both types were later reduced to their respective alcohols. Finally, the metabolites of GPS were identified as G1—erythrocentaurin; G2—gentiopicral (gentiogenal), which had been previously reported to have antibacterial, antifungal, and anti-tumor activities; G3—5-hydroxymethylisochromen-1-on; G4—5-hyroxymethylisochroman-1-on; and G5—5,6-dihydro-5-hydroxymethyl-6-methyl-1H,3H-pyrano[3,4-*c*]pyran-1-one (Figure 2).

Alongside the metabolism of GPS in the digestive tract, several studies on the intestinal absorption of the compound of interest were performed. The uptake, transepithelial transport, and efflux of gentiopicroside, together with the mechanism of its absorption, was explored in HPLC-based experiments on Caco-2 cells, conducted by Huang et al. [9]. The effects of time, temperature, and P-glycoprotein (P-gp) inhibitors on the absorption of GPS were tested in this human intestinal epithelial cell model. The authors claimed that the absorption of GPS was mainly via a passive diffusion process, and was correlated positively to time and negatively to temperature. There was no significant change between different concentrations of GPS. The inhibitors of P-gp, such as cyclosporine A and verapamil, significantly enhanced the uptake and transport of GPS, and, as expected, P-gp had strong efflux effects on the absorption of GPS.

The stability of GPS in the intestinal fluids was investigated *in vitro* in a HPLC-based study by Chen et al. [10]. It was concluded that, after 48 h of incubation, GPS was degraded by 34.4%. The degradation profile of GPS in gastric contents and mucosa homogenates, small intestinal contents, intestinal mucosa homogenates, and liver homogenates was similar to that in the aqueous buffer. After 12 h, only 20% of the initial concentration of GPS was detected in the large intestine’s content, and after 24 h, as much as 53% was detected in plasma. GTP was stable in the stomach, small intestine, and liver; however, the blood and large intestine were possible sites where GPS could be degraded.

## 4. Pharmacological Properties of GPS and Its Derivatives—*In Vitro* Studies

**Figure 3:**
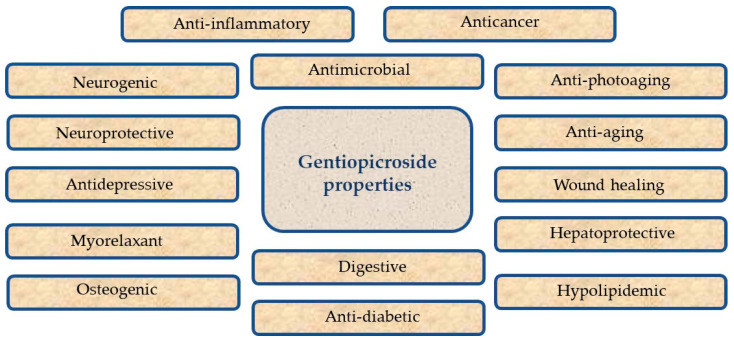
Biological properties of gentiopicroside.

Vast traditional applications of GPS-containing plants encouraged the researchers to study the biological potential of GPS more extensively and broadly. A multitude of scientific reports denoting the marked biological potential of this secoiridoid have been published. Below, the pharmacological potential of the compound that were proved in *in vitro* tests are reviewed. Some of the major biological activities of GPS are summed in Figure 3.

### 4.1. Anti-Inflammatory Activity of GPS and Its Semi-Synthetic Analogues

GPS, isolated from *Gentiana officinalis*, was tested (25, 50, and 100 μg/mL) for its anti-inflammatory properties by Zhang and co-investigators [11] in a mouse macrophage cell line (RAW 264.7) stimulated with lipopolysaccharide (LPS). The *in vitro* results showed that GPS inhibited nitric oxide (NO), prostaglandin E2 (PGE2), and interleukin-6 (IL-6) production. The overproduction of NO and PGE2 was associated with the overexpression of inducible nitric oxide synthase (iNOS) and cyclooxygenase-2 (COX-2) in cells, so GPS could inhibit the expression of iNOS and COX-2. The molecular docking of COX-2 and iNOS by GPS confirmed a satisfactory anti-inflammatory activity of the compound.

In another study in the J774A.1 cell line, increasing concentrations of GPS (1–3–10 μM/24 h) exerted anti-inflammatory activity by reducing the expression of COX-2 [12]. Also, a positive binding with cyclooxygenase-2 (COX-2), alpha-1-antichymotrypsin (AATC), and alpha-1-acid glycoprotein (orosomucoid, ORM) emerged from the computational experiments, and the outcomes from the promising interaction with COX-2 were confirmed using a Western blot.

In the study of Chang et al. [13], GPS promoted cell survival and suppressed inflammatory cytokines’ (TNF-α, IL-1β, and IL-8) production and restored the expression of IL-10, which was reduced by ethanol (ELISA). Also, the regulation of matrix metallopeptidase MPP-10 expression and pERK1/2 signaling were involved in the GPS mechanism of action, which suggests that GPS might be considered a promising therapeutic drug in ethanol-induced gastritis.

The anti-inflammatory properties of GPS were helpful in soothing inflammatory conditions in the studies of Wang et al. [14]. As a result, it was found that, *in vitro*, GPS reduced the inflammatory cytokine production of primary bone marrow-derived macrophages (BMMs) stimulated by (LPS)/IFN-γ. The secoiridoid prevented LPS/IFN-γ-induced inflammatory cytokine production by macrophages through the NF-κB (nuclear factor kappa) signaling pathway *in vitro*, and might exert a protective role by suppressing pro-inflammatory cytokine production by macrophages.

Zhang et al. [15] investigated the effect of GPS (isolated from *G. macrophylla*) on the expression of inflammatory factors in TNF-α-stimulated human fibroblast-like synoviocytes (RA-FLS) and evaluated the mechanism involved.

The treatment with GPS significantly and dose-dependently decreased the levels of IL-1β and IL-6 mRNAs, and down-regulated the expression of p-p38MAPK and NF-κB-p65 proteins in the TNF-α cell group. The treatment with 5–25 μM of GPS did not affect the number of viable cells in the MTT assay; however, at 50 and 100 μM, the number of viable cells significantly increased.

In another study, Wang et al. [16] found that GPS was able to suppress the TNF-α-induced proliferation and migration of RA-FLS cells. This suppression was attributed to the ability of GPS to block NOD-like receptor protein 3 (NLRP3), apoptosis-associated speck-like protein containing a CARD (ASC), and caspase-1, thereby disrupting the activation of the NLRP3 inflammasome. Consistent with such suppression, GPS led to a significant decrease in IL-1β secretion by the treated cells. The reduction in NLRP3 inflammasome activation was also associated with a decrease in the activation of nuclear factor (NF-κB), the production of reactive oxygen species (ROS), and the expression of inflammatory IL-6.

**Figure 4:**
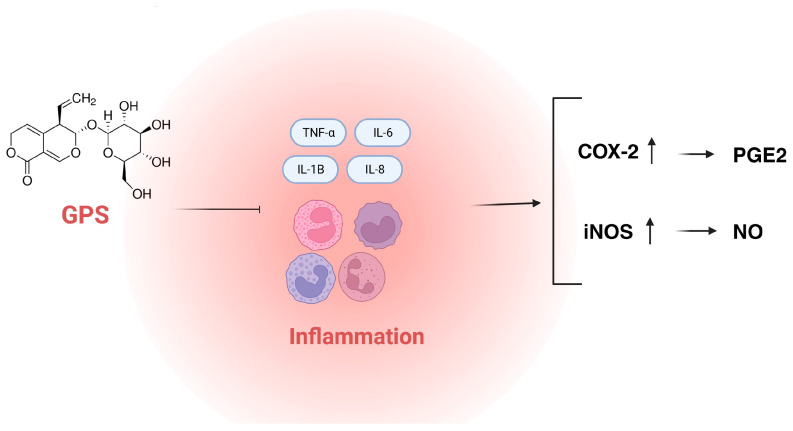
The main targets for anti-inflammatory activity of GPS (COX-2—cyclooxygenase 2; IL-1β—interleukin 1β; IL-6—interleukin 6; IL-8—interleukin 8; iNOS—inducible nitric oxide synthase; GPS—gentiopicroside; TNF-α—tumor necrosis factor α; PGE2—prostaglandin E2; NO—nitric oxide).

Due to the presence of a sugar moiety in the GPS structure, this compound is highly hydrophilic. This fact explains its reduced oral bioavailability, quick metabolism, and short biological half-life, and therefore, its limited efficacy. In their experiments, Zhang et al. [17] attempted to reduce the compound’s polarity and keep the biological activity of GPS simultaneously. The researchers introduced hydrophobic cyclic acetals into the structure of GPS to enhance its lipophilicity, aiming to obtain 26 novel derivatives of GPS with excellent anti-inflammatory activity. The most potent compound (P23) with the 4-difluoromethoxyphenyl moiety was more active than GPS, and the inhibitory potential of P23 (57.26%) exceeded that of the positive control drug celecoxib (46.05%), tested at dose of 0.28 mmol/kg. Also, other derivatives substituted with NO_2_, F, Cl, CH_3_, CF_3_, and OCF_2_H showed a satisfactory activity. It was concluded that the *para*-substitution with electron-withdrawing groups might be beneficial for obtaining the anti-inflammatory activity of GPS derivatives. Their mechanism of action was possibly associated with the down-regulation of inflammatory cytokines NO and PGE2 via the production of IL-6 and the suppression of iNOS and COX-2. These compounds may represent a novel class of selective COX-2 and iNOS inhibitors as new anti-inflammatory agents. Figure 4 presents the main targets for the anti-inflammatory activity of GPS.

### 4.2. The Effect of GPS on Collagen Production

The wound-healing properties of GPS, both alone and as the main component of a plant extract (*Gentiana lutea* ssp. *symphyandra*), were evaluated in comparison to dexpanthenol. The influence of GPS on collagen production, mitotic ability, and cell biological changes were observed using microscopy; Öztürk et al. [18] confirmed that GPS contributes to the wound-healing ability of gentian by increasing the mitotic ability and delaying the cell death of threatened cells. GPS (0.4 and 2 µg/mL) was also effective in the stimulation of collagen production, which was shown on cultured chicken embryonic fibroblasts from fertilized eggs.

### 4.3. Promotion of the Osteogenic Effect by GPS in Bone Mesenchymal Stem Cells

Jiang et al. [19] confirmed the induction of osteogenic differentiation in bone mesenchymal stem cells (BMSCs) by GPS (10–40 μM) that was a result of an increased mRNA expression of the osteogenic genes. Also, the expression of osteogenic proteins, namely Runx2 (runt-related transcription factor 2), OCN (osteocalcin), and BMP2 (bone morphogenetic protein 2), was promoted by GPS and reached a maximum for the 40 μM concentration. The following *in vitro* results showed that GPS enhanced the activity of ALP (alkaline phosphatase), increased the number of calcified nodules in BMSCs, and up-regulated the osteogenic factors (Runx2, OSX, OCN, OPN, and BMP2), which could be considered beneficial in osteoporosis treatment. GPS was not cytotoxic to BMSCs at the tested concentrations.

### 4.4. Neurogenic and Neuroprotective Activity of GPS

Chiba et al. [20] tested the ability of several secoiridoids, including GPS, to exhibit neurotrophic activity (contribute to the growth, survival, and function of brain neurons) in PC12h cells—a subclone of rat pheochromocytoma cells. GPS (1, 5, 10, 20, 50, and 100 µM) obtained from the whole plant of *Swertica japonica*, induced significant neurite outgrowth in PC12h in a dose-dependent manner. No toxic effects were observed for GPS (100 µM/2 days). As compared to other secoiridoids, GPS was found to be the most active. Structure–activity studies revealed that mono-glucosides of secoiridoids were more active than di-glucosides.

GPS was also proved to exhibit neuroprotective properties. The anti-apoptotic effects of GPS in neonatal rat hippocampal neurons following oxygen–glucose deprivation and reperfusion injury was tested by Wang et al. [21] in an *in vitro* model of ischemia and reperfusion. In their studies, pre-treatment with GPS (10, 20, 40 mg/L; 24 h before oxygen–glucose deprivation) significantly attenuated neuronal damage, and decreased the neuronal apoptosis rate (which was visualized through Hoechst 33342 staining) and the nerve cell lactate dehydrogenase (LDH) leakage rate. GPS at the highest tested dose effectively down-regulated the expression level of caspase-3 and Bax in mRNA and protein, and up-regulated the expression level of Bcl-2. Through these mechanisms, GPS pre-conditioning prevented neurons from oxygen–glucose deprivation and reperfusion injury.

### 4.5. Lifespan-Prolonging Activity of GPS

Liu et al. [22] investigated whether GPS isolated from *Gentiana rigescens* Franch, a traditional Chinese medicine, prolonged the replicative and chronological lifespans of yeast (K6001 and YOM36-GFP-Atg8 yeast cells). These organisms are used as a simple model that can be amended to genetic and molecular manipulation in studies on aging. As was found, GPS increased the survival rate of yeast under oxidative stress conditions; enhanced the activities of catalase, superoxide dismutase, and glutathione peroxidase; and decreased the levels of reactive oxygen species and malondialdehyde (MDA—the product of the lipid peroxidation of polyunsaturated fatty acids). Also, GPS (at 1 μM) significantly induced autophagy and mitophagy in yeast and extended the replicative lifespan through the regulation of mitophagy that requires the *ATG32* gene. It was proven in the PCR analysis that *ATG32* gene levels were increased by GPS (at 1 and 3 μM). In light of these findings, GPS was found to significantly prolong the replicative lifespan of K6001 and increase the survival rate of YOM36-GFP-Atg8 yeast.

### 4.6. The Effect of GPS on the Metabolism of Glucose and Lipids

The influence of GPS on gluconeogenesis was investigated *in vitro* by Yang et al. [23]. The analysis performed on human liver cells (L02) isolated from healthy adults (insulin resistant—IR; normal—NC) concluded that GPS stimulated glucose uptake with a stronger effect on IR than NC cells. This compound also suppressed gluconeogenesis in both types of cells, which indicates that GPS exerts insulin receptor-independent effects. Moreover, GPS regulated gluconeogenesis via the AKT-FoxO1 pathway by activating the phosphorylation of AKT (serine/threonine-specific protein kinase), which down-regulates the transcriptional activity of FoxO1 (Forkhead box protein O1, transcription factor), leading to an inhibited expression of key regulatory enzymes of gluconeogenesis, namely glucose-6-phosphatase (G6Pase) and phosphoenolpyruvate carboxykinase (PEPCK). GPS did not exert cytotoxic effects on L02 cells up to a concentration of 5 mmol/L (in cell viability tests), and the maximum concentration of the compound was set at 100 µmol/L.

Other studies have underlined that the abnormalities in lipid and glucose metabolism occurring in type 2 diabetes (T2DM) could be ameliorated by GPS. Considering the key role of the fibroblast growth factor receptor 1/phosphatidylinositol 3-kinase/protein kinase B (FGFR1/PI3K/AKT) pathway in T2DM, Xu et al. [24] explored the possible mechanism of GPS on lipid and glucose metabolism through its effects on the FGFR1/PI3K/AKT pathway in human hepatocellular carcinoma (HepG2) cells. GPS at concentrations lower than 320 µM showed no cytotoxicity in the tested cells. Starting from 20 µM, GPS promoted glycogen synthesis and glucose consumption; induced glycogen storage and glucose consumption; and reduced lipid accumulation in PA (0.25 mm of PA (palmitic acid)/24 h)-induced HepG2 cells. The compound ameliorated glucose and lipid metabolism disorders through the activation of the FGFR1/PI3K/AKT pathway.

In further experiments conducted by Xiao et al. [25] on HepG2 cells that were treated with GPS (5–320 µmol/L/24 h), it was found that 80 µmol/L of GPS showed similar effects as 250 µmol/L of metformin in an increased glucose utilization. The treatment resulted in an improved glycolipid metabolism with no toxicity. According to the authors, GPS inhibited the interaction between adipoQ receptor 3 (PAQR3) and the PI3K catalytic subunit to restore the PI3K/AKT signaling pathway. GPS directly bound with amino acids of the PAQR3 NH_2_ terminus (including Leu40, Asp42, Glu69, Tyr125, and Ser129, which is an important binding site for GPS). Also, GPS ameliorated high-fat diet (HFD) and palmitic acid (PA)-induced hepatic insulin resistance by promoting PI3K/AKT axis activation. These findings provide a rationale for the potential application of GPS to restore insulin sensitivity in diabetes.

In the experiments conducted by Xu et al. [26] on HG-stimulated NRK-52E cells (rat kidney epithelial cells cultured with 30 mM of high glucose), GPS (12.5, 25, and 50 μM) inhibited renal tubulointerstitial fibrosis (TIF—pathological changes that may occur in diabetic nephropathy), which may be related to the glucose-lowering activity of GPS. The activation of casein kinase 2 (CK2) is closely linked to the body disturbance of carbohydrate metabolism and inflammatory reactions [27]. The secoiridoid may also directly inhibit the CK2/NF-κB inflammatory signaling pathway via angiotensin II type 1 receptor (AT1R) to ameliorate TIF in diabetes. As was confirmed, the effects of GPS that were observed *in vitro* in HG-stimulated NRK-52E cells were reversed by AT1R over-expression.

**Figure 5:**
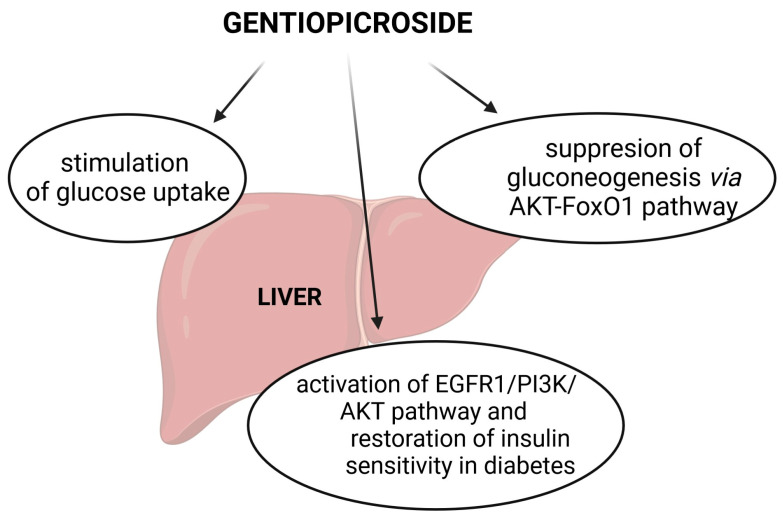
The effects of GPS on the metabolism of glucose (EGFR1—epidermal growth factor receptor 1; FoxO1—Forkhead box O1; PI3K—phosphoinositide 3-kinase).

The effects of GPS on glucose metabolism are summed up in Figure 5.

### 4.7. The Effect of GPS on Cytochrome P450 Activity

Pooled human liver microsomes (*n* = 16, mixed gender) were used for the investigation of the ability of GPS to inhibit the major drug-metabolizing P450 enzymes. Human cytochrome P450 (CYP) enzymes, as membrane-bound hemoproteins, play important roles in the detoxification of drugs, cellular metabolism, and homeostasis. In their *in vitro* study, Deng et al. [28] used human primary hepatocytes obtained from a 34-year old Mongolian man, who was homozygous for the CYP2D6 wild type of CYP450. To evaluate GPS (absence or presence of GPS at concentrations from 1.0 to 1000 µg/mL) as a direct-acting inhibitor, pooled human liver microsomes were incubated with P450 marker substrates, and the reactions of *O*-dealkylation (Phenacetin); 6β-, 4′-, and 6- hydroxylation (Testosterone, Diclofenac, Coumarin, S-Mephenytoin, Chlorzoxazone); and *O*-demethylation (Dextromethorphan) were monitored via LC-MS.

The direct inhibition of CYP2A6 and CYP2E1 activity by GPS was observed in a concentration-dependent manner. The CYP2A6 enzyme was inhibited by GPS to the largest extent (80% activity reduction) with IC_50_ = 21.80 µg/mL, which was close to the value observed in clinical studies (19.50 µg/mL). GPS had little effect on CYP2E1 or CYP3A4, which have been reported to be involved in the mechanism of hepatitis therapy.

Several studies have reported that AMP-activated protein kinase (AMPK) is a lipid metabolism-regulating kinase with an important role in alcoholic liver disease (ALD) pathogenesis. AMPK activity decreases during ethanol intake, and simultaneously, ethanol increases acetyl-CoA carboxylase (ACC) activity. Consequently, this change exacerbates the imbalance in lipid metabolism. Therefore, inhibiting lipid accumulation or promoting lipolysis may prevent liver steatosis from further developing into liver fibrosis (which results in the loss of liver function). Hepatic stellate cells (HSCs), normally rich in vitamin A, when targeted by ethanol or viruses, lose vitamin A and are activated. This results in the secretion of large amounts of liver fibrosis markers, such as collagen I, smooth muscle α-actin (α-SMA), and transforming growth factor-β (TGF-β). Thus, controlling the activation of HSCs is a key target of reversing liver fibrosis.

Yang et al. [29] tested whether and how GPS could hold back the progression of liver steatosis to liver fibrosis. Also, the inhibitory capacity of HSC activation by GPS (25, 50, and 100 µM) in a human cell line (LX-2), as well as the amelioration of lipid accumulation in mouse AML12, was evaluated. It was found that GPS pre-treatment decreased collagen I, and α-SMA was upregulated by TGF-β in LX-2 cells. This indicates that GPS could reverse the initiation of hepatic fibrosis during chronic alcoholic liver disease. GPS inhibited lipid accumulation by promoting lipid oxidation and inhibiting lipid synthesis.

It was also found by Xiao et al. [30] that GPS inhibited the production of inflammatory adhesion molecules (ICAM-1 and VCAM-1) and the intercellular matrix (fibronectin and collagen IV) in the liver, revealing a protective role of GPS in fatty liver fibrosis.

### 4.8. Myorelaxant Activity of GPS

Rojas et al. [30] proved that GPS (0.01–100 µg/mL), isolated from the aerial parts of *Gentiana spathacea*, inhibited, in a concentration-dependent manner, the spontaneous contractions of isolated guinea pig ileum. GPS significantly reduced the contractions induced in the ileum by BaCl_2_ and KCl (52.5% and 32.2% inhibition, respectively), and produced a moderate inhibition of the contractions induced by acetylcholine and histamine (19.0% and 22.6%, respectively). These findings suggest that GPS may block the influx of extracellular Ca^2+^ to smooth muscle cells. However, it was not excluded that GPS may also compete with Ca^2+^-binding proteins such as calmodulin.

### 4.9. Antibacterial Potential of GPS

As was reported by Kumasaramy et al. [31], GPS from the aerial parts of *Centaurium erythrea* inhibited the growth of 12 out of 17 pathogenic bacterial species (bacterial concentration of 5 × 10^5^ cfu/mL was tested, using a 96-well microplate-based broth dilution assay). The obtained minimum inhibitory concentrations (MICs) were between 6.3 × 10^−3^ and 1.0 × 10^−1^ mg/mL. GPS was the most active against *Serratia marcescens* (6.3 × 10^−3^ mg/mL), with a weak activity against *Staphylococcus aureus* (1.0 × 10^−1^ mg/mL), and was not active against pathogenic bacteria such as *S. aureus* (MRSA), *S. epidermis*, *E. coli*, and *Salmonella goldcoast*.

#### Antibacterial Activity of Nanoparticles with GPS

Almukainzi et al. [32] prepared a nanoform of GPS that was composed of PLGA NSs (Poly-lactic-co-glycolic acid nanospheres; NSs) nanocarriers to enhance its solubility and improve its oral absorption. GPS-PLGA chemical and physical interactions have been analyzed using Fourier-transform IR spectroscopy (FTIR) and differential scanning calorimetry (DSC). The optimum GPS-PLGA NSs have been chosen for an antimicrobial study to investigate its inhibitory action on *Staphylococcus aureus* compared with GPS unloaded on NSs. The optimum GPS-PLGA NSs (F5) with well-controlled particle sizes (250.10 ± 07.86 nm), a relatively high entrapment efficiency (83.35 ± 5.71), and the highest % cumulative release (85.79 ± 8.74) increased the antimicrobial activity, as they exhibited a higher inhibitory effect on bacterial growth than free GPS.

### 4.10. In Vitro Antiviral Activity of Semi-Synthetic GPS Derivatives

The synthesis and pharmacological evaluation of a series of GPS derivatives as potential antiviral inhibitors was undertaken by Wu et al. [33]. Synthesized compounds were biologically evaluated for their inhibition of an influenza virus and their anti-HCV (hepatitis C virus) activity *in vitro*. Some of the GPS derivatives, such as 11a (2′,3′,6′-tri-*O*-benzoyl-4′-*O*-methylsulfonyl gentiopicroside), 13d (4′-fluoro-4′-deoxygentiopicroside), and 16 (2′,3′,6′-Tri-*O*-benzoyl-4′,5′-olefin gentiopicroside), showed interesting anti-influenza virus activity with IC_50_ at 39.5 mM, 45.2 mM, and 44.0 mM, respectively. No significant anti-HCV activity was found for all of the GPS derivatives. The preliminary results indicate that the modification of the sugar moiety of GPS was helpful for enhancing its anti-influenza activities.

### 4.11. Oxidative Stress-Reducing Potential of GPS

Abnormal lipid metabolism triggers the oxidative stress condition and has an influence on the development of cardiovascular and cardiac diseases, type 2 diabetes mellitus, and chronic kidney disease [34]. The nuclear erythroid 2-related factor 2 (Nrf2) is known as the “main regulator” of the antioxidant response that regulates the expression of hundreds of genes. It can regulate oxidant defense systems, including the expression of stress response proteins, HO-1; the synthesis of reducing factors, such as glutathione (GSH); and the promotion of the catabolism of peroxides and superoxides, such as superoxide dismutase (SOD). Nrf2 activation can be controlled by the phosphatidylinositol 3-kinase PI3K/AKT pathway. The protective effects of GPS on lipid accumulation and oxidative damage was investigated by Jin et al. [34] in free fatty acid (FFA)-induced HepG2 cells of the wild type (WTHepG2) and Nrf2^-/-^HepG2 to analyze if GPS could ameliorate lipid accumulation through Nrf2 activation. WTHepG2 cells were exposed to 1 mM of FFA (oleate/palmitate = 2:1) for different time points from 0 h to 24 h. To demonstrate the cytotoxicity of GPS, HepG2 cells were incubated with different concentrations of GPS for 24 h. Because 500 μM of GPS significantly decreased cell viability, the influence of GE was tested in the range of 0–500 µM (0, 6, 12, 18, and 24 h). Cell-counting Kit-8 assays; Oil Red O staining; a Western blotting analysis; the extraction of nuclear and cytosolic proteins; a biochemical index assay; and the measurement of triglyceride (TG), total cholesterol (TC), alanine transaminase (ALT), aspartate aminotransferase (AST), and malondialdehyde (MDA) in cell lysates were employed to explore the mechanisms by which GPS exerts a protective effect on FFA-induced HepG2 cells. It was found that GPS significantly regulated the activation of the PI3K/AKT signaling pathway, the Nrf2 antioxidant pathway, and peroxisome proliferator-activated receptor α (PPARα). GPS also inhibited sterol regulatory element-binding protein-1c (SREBP-1c) expression in FFA-induced HepG2 cells. Through the upregulation of the Nrf2 antioxidant pathway, GPS can alleviate oxidative damage and lipid accumulation and strongly protect WTHepG2 cells from both processes.

### 4.12. Anti-Proliferative Potential of Gentiopicroside

#### 4.12.1. Ovarian Cancer

Ovarian cancer encompasses many types of neoplasms with distinct molecular and clinic–pathological features as well as prognoses [35,36]. Ovarian cancer is the seventh most common cancer with five-year survival rates below 45% and the eighth most common cause of cancer death in women worldwide. The number of cases diagnosed every year is increasing [37]. It has been demonstrated that gentiopicroside exhibits significant anti-cancer activity against SKOV3 ovarian cancer cells. GPS (IC_50_ = 20 μM) displays an anti-proliferative effect and inhibits the growth of SKOV3 cells in a dose-dependent manner. Moreover, GPS leads to apoptosis in a concentration-dependent fashion. It has also been detected that the number of cells in the G2 phase of the cell cycle increases after a 40 µM GPS treatment, leading to cell cycle arrest. Additionally, GPS (20 μM/24 h) reduces the migration and motility of SKOV3 ovarian cancer cells in a dose-dependent manner. The inhibition of SKOV3 cancer cell migration suggests that GPS potentially restrains the metastasis of cancer cells. The induction of the mitochondrial apoptosis pathway, cell cycle arrest, and the inhibition of cancer cell migration and invasion indicate that GPS can be considered a promising molecule for the therapy of ovarian cancer [38].

Other authors have demonstrated that GPS can inhibit cell growth in OVCAR-3 ovarian cancer cells through the NF-κB (nuclear factor kappa-light-chain-enhancer of activated B cells) signaling pathway, the mediation of apoptosis, and altering the expression of several apoptosis-related proteins, as well as the disruption of mitochondrial transmembrane potential. It was revealed that GPS treatment led to the up-regulation of cytochrome c, cleaved PARP-1 (poly (ADP-ribose) polymerase 1), and caspase-3 and -9, as well as the down-regulation of Bcl-2 (B-cell lymphoma 2) and NF-κB protein expression in a dose-dependent manner. All these proteins play a pivotal role in the induction of apoptosis in cancer cells [39].

#### 4.12.2. Cervical Cancer

Despite the fact that screening programs are widespread, cervical cancer remains the third most common cancer in developing countries [40]. It has been demonstrated that GPS exhibits anti-cancer activity against HeLa cervical cancer cells through the induction of apoptosis, the inhibition of cell growth and migration, and cell cycle arrest via the regulation of the MAPK (mitogen-activated protein kinases)/AKT signaling pathway. GPS shows an anti-proliferation effect on HeLa cancer cells, in contrast to normal human umbilical vein endothelial cells (HUVECs) at the same dosage of administration. Moreover, an increased number of cells with a bright nuclear condensation and fragmentation of nuclei have been observed in HeLa cells stained with Hoechst 33342 after GPS treatment. GPS has also caused comet formation in HeLa cells. Furthermore, the tail area, tail DNA percentage, tail DNA, tail moment, and tail length were significantly increased, which is correlated with apoptosis after GPS treatment. It has been shown that the mitochondrial pathway involves gentiopicroside-induced apoptosis in HeLa cells. GPS has decreased JC-1 aggregates and significantly enhanced the creation of monomers, suggesting that the mitochondrial membrane potential decreased (which is a landmark event in the early phase of apoptosis). The level of cleaved caspase-8 was decreased, whereas the expression of cytochrome c, cleaved caspase-9 and -3, and AIF (apoptosis-inducing factor) in the cytoplasm were increased. Dose- and time-dependent cell cycle arrest at the G2/M phase was observed in HeLa cells after GPS treatment. Moreover, the protein expression of CDK1 (cyclin-dependent kinase 1) and cyclin-B1 was decreased, while p53 and p21 protein expression was increased after GPS treatment. Moreover, GPS strongly alters the AKT- and MAPK-related proteins, which inhibits the phosphorylation of p38, Erk1/2, and AKT, while the phosphorylation levels of c-Jun and JNK (c-Jun N-terminal kinase) were significantly elevated. The protein expression of MMP-2 (matrix metalloproteinase-2) was significantly downregulated, while the level of MMP-9 (matrix metalloproteinase-9) was not altered. All these results suggest that GPS inhibits the migration and effectively suppresses the metastatic potential of HeLa cells by down-regulating MMP-2 expression [41].

#### 4.12.3. Gastric Cancer

Gastric cancer is one of the most widespread malignant tumors in the digestive tract [42]. Gastric adenocarcinoma is the fifth most common and the third most lethal type of cancer worldwide. Huang et al. [43] tried to find the molecular mechanisms and potential targets of GPS in gastric cancer using a computational methodology. They also used molecular docking and *in vitro* assays to validate the effect of GPS in gastric cancer cells. It has been revealed that GPS can result in multiple effects in gastric cancer cells by regulating multiple pathways. Huang et al. found 135 targets for GPS in gastric cancer. A docking analysis demonstrated that GPS has a good binding activity to cyclin-D1 (CCND1). The authors selected AKT, p38, CCND1, and CCNE1 as the potential targets of GPS. The level of expression of hosphor-p38 (p-p38) protein was significantly lower after GPS treatment compared with the control group. However, the expression of CCND1, CCNE1, and p-AKT proteins was significantly higher. The PI3K/AKT signaling pathway is one of the most frequently activated signal transduction pathways in cancer biology and is involved in the cell proliferation, growth, migration, apoptosis, cell cycle, and angiogenesis processes. It has been also reported that GPS was selectively active against human gastric cancer cells in contrast to normal gastric epithelial cells [44].

#### 4.12.4. Hepatocellular Carcinoma

Hepatocellular carcinoma is one of the most common cancers worldwide, responsible for approximately one million deaths every year, mostly in sub-Saharan Africa and the Far East. Hepatocellular carcinoma very often presents at an advanced stage and has a very poor prognosis [45]. GPS and five other kinds of traditional Chinese medicine extracts can lesion SMMC-7721 human hepatocellular carcinoma cells; however, the exact mechanism of action is not yet known [46].

#### 4.12.5. Other Types of Cancer

GPS was proven to exhibit anti-cancer effect when tested in other cancer models as well. In the manuscript by Antoniadi et al. [47], the anti-proliferative action of GPS was reported for the following cancer cell lines: glioblastoma (T98G), lung (A549, NCI-H1299), rhabdomyosarcoma (TE671), breast (MCF7, MDA-MB-468), and colon (HT-29, DLD-1) cancer cells, as well as and normal colon epithelial cells (CCD 841 CoTr).

**Figure 6:**
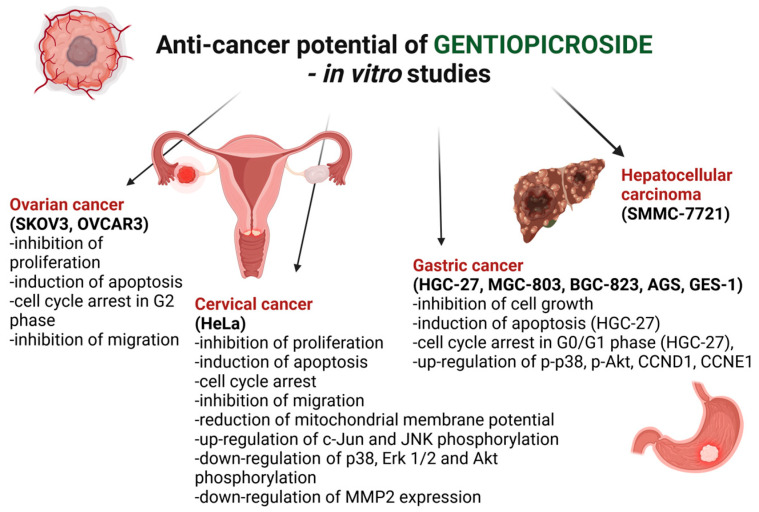
Mechanisms of the anti-cancer activity of GPS in various cancer cell lines.

The major anti-cancer properties of GPS that have been proven *in vitro* are listed in Figure 6.

## 5. Pharmacological Properties of Gentiopicroside *In Vivo*

According to scientific databases, recent years have brought the development of in vivo studies on gentiopicroside. Our search performed in the Scopus database showed that the number of scientific articles that cover the topic of the *in vivo* testing of GPS reached eight annually, whereas, prior to 2014, one article was released annually. This confirms an increasing interest in *in vivo* studies on GPS that are conducted in various directions. The compound is researched for its antimicrobial, anti-inflammatory, central nervous system-targeting, osteogenic, anti-dermatophyte, anti-diabetic, or weight-reducing properties. The detailed characteristics of the aforementioned properties are presented below and in Figure 4.

### 5.1. Anti-Inflammatory Properties of GPS In Vivo

In the study of Jia and co-investigators [48], the anti-inflammatory activity of GPS was investigated in male C57BL/6J mice that were administered GPS intragastrically at concentrations of 20 and 40 mg/kg of b.w. for a period of 30 days. As a consequence of GPS treatment, an inhibition in paw edema was observed for both tested doses; however, the therapeutic effects of the 40 mg/kg dose appeared earlier (8 days for 40 mg/kg vs. 22 days for 20 mg/kg). Both doses reduced the inflammatory infiltration, joint destruction, and bone damage thanks to the decreased serum cytokine levels (IL-1β, IL-6, and TNF-α) and suppressed CD147, p-p38, p-IκBα, MMP1, MMP2, and MMP3 *in vivo*.

The proven anti-inflammatory potential of GPS has encouraged scientists to study the properties of its semi-synthetic derivatives. In the study of Zhang and collaborators [15], some derivatives decreased the edema percentage by 34.17% after 7 days of administration of a dose of 0.28 mmol/kg of b.w.

The anti-inflammatory properties of GPS were also tested in a gouty arthritis model in male C57BL/6 mice [49]. The 24 h long treatment with 100 and 200 mg/kg of GPS p.o. showed a reduction in swelling, the occurrence of analgesic properties, and the inhibition of thermal hyperplasia. GPS inhibited the infiltration of neutrophils by alleviating the levels of interleukins IL-1β, IL-6, IL-18, TNF-α, caspase-1, NOD-like receptor protein 3, and ACS.

Another mechanism of anti-inflammatory action proposed by Wang et al. [18] included the GPS-based alleviation of the ROS-NF-κB-NLRP3 axis in synoviocytes, as well as the NF-κB pathway. As a consequence of a 14-day-long treatment with 200 mg/kg of GPS, a reduction in paw swelling and a decrease in the arthritis index value were observed. A histopathological analysis of joints showed reduced inflammation, bone destruction, synovial hyperplasia, and pannus formation, and no impact on body weight or spleen index, but a slight reduction in the thymus index value. GPS treatment inhibited IκBa degradation and p-IκBa and p-p65 expression levels in the synovial tissue, which suggests the joint-protective role of this natural product and confirms that its action is related to NFκB signaling.

The anti-inflammatory properties of GPS could find applications in the treatment of pulmonary fibrosis [50]. The compound administered to male SPF mice for 28 days decreased the concentration of hydroxyproline in the lung tissue and ameliorated fibrotic parameters (alveolar space narrowing, alveolar wall thickening, deposition of collagen) and inflammatory responses. The described properties occurred together with decreased levels of TNF-α, IL-1β, and TGF-β1.

GPS at a dose of 30 µM was found to exert anti-inflammatory properties in a zebrafish model of COX-2 enzyme inhibition [51]. GPS was found to inhibit the enzyme at 65 ± 5%, whereas the anti-inflammatory drug indometacin at a concentration of 0.9 µM was prone to inhibit the enzyme at 56 ± 1%.

### 5.2. GPS in the Treatment of Neuropathies In Vivo

Xu et al. [26] described the application of GPS in diabetic renal fibrosis—a symptom which is a consequence of neuropathy developed with the progression of diabetes mellitus and hyperglycemia that leads to an excessive deposition of extracellular matrix in the kidneys that disturbs their physiological functioning. The study was performed on 6-week-old male db/db mice that were intragastrically administered different doses of GPS (50, 100, and 200 mg/kg of b.w.) for 10 consecutive weeks. Valsartan (10 mg/kg of b.w.) was used as a positive control, and healthy male C57/BL6 mice as the control group. Animals treated with GPS were found to have an ameliorated metabolism of lipids and glucose that was confirmed by decreased levels of HbA1c, GSP, LDL-C, TG, and body weight at all tested doses and FBG (after 3 weeks of treatment). Also, the compound supported renal functions. The levels of creatinine, urea nitrogen, and albuminuria were significantly decreased in the treated animals. Staining tests showed an increased expansion in the lumen, glycogen accumulation in the renal cortex of the treated group, and a lowered degree of inflammation and fibrosis in the renal tubule. The latter effects were explained by an alleviated level of FN, α-SMA, and vimentin, and increased expression of E-cadherin after GPS treatment. As a result of these studies, the authors denoted that the secoiridoid suppresses the AT1R/CK2/NF-κB pathway.

According to Lu and collaborators [52], GPS decreased hyperalgesia in Sprague Dawley rats stimulated with hot, cold, and mechanical allodynia. The application of GPS in the treatment of diabetic peripheral neuropathy was suggested by the authors, as the compound was capable of restoring nerve blood flow, improving motor nerve conduction velocity and sensory nerve conduction velocity parameters, and regulating dyslipidemia. GPS influenced the expression of genes of the PPAR-γ/AMPK/ACC signaling pathway.

### 5.3. The Effect of GPS on the Metabolism of Glucose and Lipids In Vivo

GPS was also tested for its potential application in the treatment of alcoholic hepatosteatosis. The study of Li et al. [53] that was performed on male C57BL/6 mice that were treated with GPS and fed with ethanol in acute (3 days) and chronic (10 days) experiments showed several protective properties exhibited by the secoiridoid. A lower dose of 40 mg/kg of b.w. in the chronic administration prevented the increase in ALT and AST levels, as well as TG levels, in the serum and liver. The formed lipid droplets in the liver were smaller and less abundant. Also, increased LKB1 and AMPK phosphorylation levels were noted, SREBP1 protein expression was alleviated, the phosphorylation of ACC was restored, and PPARα regulatory genes were down-regulated. A protective role of GPS may be based on the inhibition of lipogenesis that occurs upon the influence of ethanol and through the elevation of lipid oxidation. GPS decreased the alcohol-induced expression of the receptors NLRP3 and P2X7, but only as a consequence of alcohol toxicity.

### 5.4. Central Nervous System-Targeting Activities of GPS In Vivo

GPS was proven to cross the blood–brain barrier and exhibit certain actions in the central nervous system. Several scientific papers describe its antidepressant activity. In the study of Liu et al. [54], who used a mouse model of reserpine-induced depression, GPS was administered intragastrically to male animals twice daily for 3 days and once on the fourth day before behavioral testing in three doses: 50, 100, and 200 mg/kg of b.w. GPS at the two highest doses decreased mechanical allodynia and immobility time in a forced swimming test and tail suspension test, and increased the traveled distance and the time in the center area in an open-field test. The observed behavioral changes in the studied animals were possibly triggered by the changes in the neuromodulator’s levels. GPS reversed the activity of reserpine that decreased the levels of norepinephrine, dopamine, and serotonin. However, when administered alone at a dose of 100 mg/kg, it did not cause any changes in neurotransmitter levels. Also, GPS reduced the intracellular levels of MDA production in the basolateral amygdala (BLA) of the treated mice; elevated the activity of catalase in their tissue, as well as Bcl-2 expression; and inhibited the activity of caspase-3 in a dose-dependent manner. The administration of reserpine led to an increased expression of the AMPA and NMDA subunits of BLA homogenates. In this study, GPS did not affect the expression of GluA1 and GluN2A subunits, but decreased the expression of GluN2B in reserpinized mice, which—according to the authors—may contribute to the attenuation of the depression dyad in tested mice.

The corticosterone-induced model of depression applied by Yao and co-investigators [55] on rats led to the recognition of the molecular mechanisms of GPS’s action, which was administered via gastric instillation at a dose of 100 mg/kg of b.w., 2 h prior to corticosterone, for 21 days. The authors proved a protective role of the compound against the steroid: the ability to inhibit apoptosis in the hippocampus that was caused by the steroid, to reduce the loss of Niss bodies, to increase the level of serotonin in the brain tissue, to elevate the expression of BDNF, and alleviate caspase 3 and Bax expression in the brain. An HPLC-MS-based chemometric analysis of the GPS-treated and untreated groups showed marked differences in the metabolite profiles between the groups. The former group was characterized with alleviated levels of sphinganine, stearoylethanolamide, and guanosine, and decreased levels of arachidonic acid, oxoadipic acid, L-phenylalanine, and thiamine. A pathway analysis provided evidence on the influence of GPS on sphingolipids, glycine/serine/threonine, and pyrimidine metabolism.

Deng et al. [56], in their study on BALB/C mice with lipopolysaccharide-induced depression that were administered with GPS (50 mg/kg of b.w. i.p.) once a day for 3 days, observed an alleviated activation of tryptophan metabolic pathways and a reduction in TNF-α and IL-1β levels in the brain (BLA) and plasma, and confirmed the expression of the GluN2B subunit of the NMDA receptor. Also, a down-regulation of indoleamine 2,3-oxygenase by GPS was noted. According to the authors, the antidepressant effect that was visualized in behavioral studies in forced swimming tests and tail suspension tests, as well as protection against the injected lipopolysaccharide, may be at least partially influenced by the ability of GPS to block different steps of the tryptophan-degrading pathway.

### 5.5. Other Activities

The secoiridoid administered orally at a dose of 50 mg/kg of b.w. for 12 weeks to mice fed with high-fat diet was proven to inhibit all key adipogenic transcription factors, like PPARγ, C/EBPα, and SREBP-1c, to down-regulate the expression of genes related to the transport of fatty acids, like the lipid uptake gene and the fatty acid (FABP4) and triglyceride (TG) transport-related gene, but also synthesis-related genes (DGAT2, FAS, SDC1) [57]. Also, the confirmed anti-inflammatory properties of GPS were expressed as regulatory towards the inflammatory genes, leading to a decreased cytokine release, which helped to reduce the weight of animals together with the visceral fat mass, decreasing the size of adipocytes.

According to Jiang et al., GPS promotes osteogenesis [19]. In a 3-month-long study on ovariectomized female C57BL/6 mice, the secoiridoid at a daily dose of 50 mg/kg of b.w. (oral gavage) promoted alkaline phosphatase activity and increased the level of osteogenic factors like Runx2, OCN, and BMP2, as well as the thickness and number of trabeculae, after the performed therapy, which was visualized in a micro-CT test. These observations provide scientific evidence on the possibility of GPS application in regulating bone metabolism.

As mentioned above, GPS shows antimicrobial potential. In the study published by Almukainzi and co-investigators [58], its antimicrobial properties were used in diabetic rats with infected wounds. GPS, together with thymoquinone, released from the constructed nanofibers led to a speedy recovery of the tissue and triggered the healing process. Another study of these authors [32] that was performed on streptozotocin-induced diabetic rats intended to histologically analyze the state of would incisions at different day intervals after the application of PLGA nanoparticles loaded with GPS. Interestingly, the tested formulation, orally administering 300 mg/kg of b.w. of GPS by increasing the bioavailability of GPS poorly dissolved in water, enhanced the antimicrobial effect in model animals (Sprague Dawley rats) in comparison with free GPS. The proposed treatment led to complete wound healing and wound closure within 12 days. Free GPS was found to induce wound-healing properties, too; however, the recovery time was longer and might have needed a more frequent administration. The regenerated tissue was normally organized, with developed mature collagen fibers, a minimal number of inflammatory cells, and physiological vasculatures.

**Figure 7:**
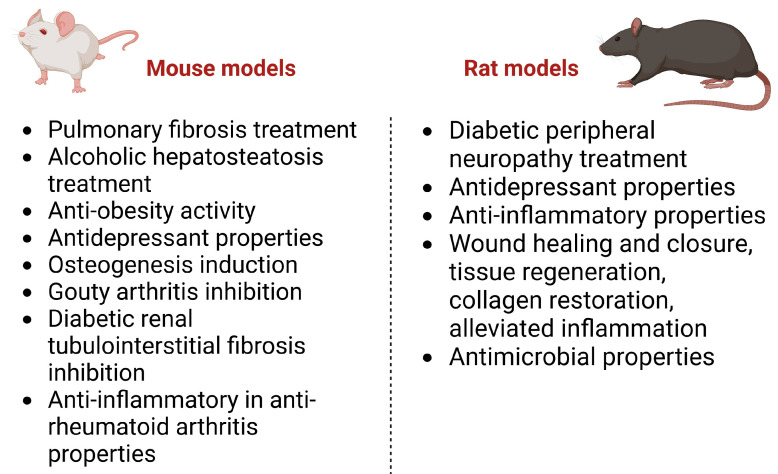
Biological effects of GPS, proven in *in vivo* studies.

Figure 7 summarizes the biological effects of gentiopicroside in *in vivo* studies conducted on animals (mice and rats).

## 6. Pharmacological Properties of Gentiopicroside: Clinical Studies

The main drawback of the GPS currently being evaluated in clinical trials is its relatively poor lipophilicity and suboptimal pharmacokinetic properties. However, only a few clinical studies related to the activity of GPS on skin have been conducted so far. A gel cream containing 65% pure GPS was administered in a single-center, randomized, double-blinded trial around the eyes of 22 Caucasian subjects of French origin aged 43–64 years old, for 14 days, twice a day, at a concentration of 147 μg/mL. The compound was obtained via a chromatographic enrichment of a hydro-alcoholic extract from *Gentiana lutea*. Alongside GPS, the sample contained polyphenols (ca. 3% of dried weight), polysaccharides (ca. 3%), and sugars (ca. 8%).

As a result of this study, no irritation of the eyelid area was noted in all volunteers, but a reduction in the average skin roughness was observed, together with skin relief in the tear trough area. Also, the redness of dark cycles was reduced in the tested subjects [59]. The obtained results show GPS as a non-irritant, mild, soothing, and skin color-correcting ingredient of cosmetics that can be used for the treatment of sensitive skin.

## 7. Possible Long-Term Adverse Effects of GPS as a Single Compound

The possible long-term adverse effects of GPS, such as genotoxic, mutagenic, and clastogenic properties (5, 10, 25, and 50 µg/mL), were assessed by Mustafayeva et al. [4]. The results present evidence that GPS exerts genotoxic activity against both prokaryotic and eukaryotic cells. The specific positive response obtained with the TA102 tester strain suggested the involvement of oxidative DNA lesions, probably due to the presence of hydroxyl groups that may produce oxygen singlets. When GPS was used as the one component of the plant extract, no toxicity was observed; however, to gain more information, a long-term study should be undertaken. However, the comparison between GPS’s molecular structure with predictive models in computational databases (Bioinformatics 2000, 2006) revealed a weak structural similarity to established genotoxic and mutagenic agents.

To analyze the toxicity of GPS, Sobot et al. [60] conducted an experiment on peripheral blood mononuclear (PBMC) cells. A cell culture was tested for viability (Tb and XTT viability assays) and genotoxicity (alkaline comet assay), employing *Gentiana lutea* root water extract (1:5, m/V ratio) and single GPS (20–130 µM), respectively. GPS at the concentration of 50 µM reduced the number of viable unstimulated PBMC cells by 25% and significantly reduced the number of viable PHA-stimulated PBMCs compared to the control, ranging from 13 to 16%. Interestingly, the GPS concentration in the gentian root extract was 10 times higher than in the GPS treatment, exerting a significant toxic effect, indicating that the single isolated compound has different effects to when it is one of the main components in a crude extract.

## 8. Conclusions and Future Perspectives

With food products, dietary supplements, or medicines, we deliver a majority of various non-nutrients, including bioactive or toxic compounds [61]. As was summarized in this article, gentiopicroside is a multifunctional metabolite that could exert different pharmacological effects, proven in *in vitro* and *in vivo* models. It interacts with different receptors present in various tissues and organs; however, the detailed mechanisms of its action still need to be extensively studied. Taking into account the various possible effects of GPS in the body, and the fact that the barriers to the action of this compound on some tissues still seem to be unresolved, new therapeutic approaches taking into account the possible better bioavailability of GPS could be beneficial. The health benefits of GPS cannot be excluded if new drug formulations (such as microencapsulation, nanoparticles, liposomes, or micelles) would be implemented to increase the bioavailability of this compound and its distribution to the target organs and tissues [31,62]. New forms of GPS would enable clinical studies on GPS, allowing for the establishment of final bioactivity results, efficacy, and potential toxicity/safety studies, especially in neuroinflammatory and metabolic disorders.

As mentioned in different scientific articles, many plants used as medicines very often interact with the human body through a bitter taste, which is one of the most important signals that a person receives from the external environment informing about potentially dangerous changes that result in the dysregulation of the body’s homeostasis.

Type 2 taste receptors (T2Rs) are G-protein coupled receptors (GPCRs) that were first identified in the mouth as receptors of bitter taste. It has been assumed that, responding to the pressure of food selection, different species have evolved with different numbers of T2Rs. Recently, there has been a growing interest in assessing the role of T2Rs in humans [62,63,64], where the T2R gene family consists of 25 functional receptor-encoding genes, many of which are polymorphic. We already know that these receptors, which have a relatively simple structure (compared to the receptors representing other tastes, such as sweet, sour, or salty) [65], are widely distributed in the human body, also beyond the tongue and oral cavity. In fact, T2Rs have been found in many different tissues and organs, e.g., in the digestive tract, immune system, nervous system, and pulmonary tract [63,64], and throughout the body, T2Rs are sentinels that monitor environmental challenges and coordinate defensive endocrine, behavioral, and immunological responses [65].

Different studies performed on bitter compounds show the marked potential of these molecules in the treatment of different diseases. One of the active stimulators of bitter taste receptors is amarogentin, which is known to be one of the most bitter compounds. The proven pharmacological properties of amarogentin through bitter taste receptors, like vascular muscle or airway smooth muscle relaxation, increased intracellular calcium release, increased extracellular ATP release, or the inhibition of calcium channel-related vasoconstriction, could be the effects of GPS’s action [66]. As this topic is new in the scientific literature and only a few models have been elaborated to study the role of bitter taste receptors, the properties of GPS have not yet been sufficiently studied. There is only one publication by Behrens et al. [67] on gentiopicroside from *Gentiana lutea*. The compound at the tested dilutions from 1:1000 to 1:10,000 did not exert effects on the 25 T2Rs. It is, however, still challenging to understand the role of T2Rs’ polymorphism in the body and their interactions with naturally occurring compounds, to elaborate suitable assays that can effectively determine the active compounds and track their impact on the receptors, and to investigate the role of the bioavailability of a compound vs. the impact on the receptors. Also, there may be a correlation of T2Rs with diseases such as asthma, cardiovascular disease, glucose and insulin homeostasis, Parkinson disease, colorectal cancer, and thyroid problems [63]. All of these may be the target of GPS and an area of future research. However, some new models will be necessary to design and make accessible detailed studies on the interaction of GPS with tissue-specific receptors, which is definitely worth trying.

Certainly, new solutions introduced to *in vitro* studies, like 3D cell cultures or organoids, could be an interesting model to evaluate of the biological activity of GPS.

In the end, it should be mentioned that, with this review of the scientific literature on gentiopicroside, we would like to encourage researchers to explore the possible effects of GPS and its interactions with various target tissues, hoping for a better understanding of the role of bitter compounds and their benefits on human health that may be discovered in the future.

## Data Availability

Not applicable.
